# Diagnostic Value of Perfusion MR Imaging as a Potential Ancillary Test for Brain Death

**DOI:** 10.5334/jbsr.2055

**Published:** 2020-09-18

**Authors:** Utku Mahir Yildirim

**Affiliations:** 1 İzmir Medicalpark Private Hospital, TR

**Keywords:** brain death, perfusion MR, DSC-MR

## Abstract

**Objective:**

The purpose of this study is to determine the potential role of dynamic susceptibility contrast (DSC) magnetic resonance (MR) perfusion imaging in diagnosing brain death.

**Materials and Methods:**

The study population was composed of 61 subjects (the Glasgow Coma Scale [GCS] score was 3 for all subjects), and 26 subjects were assigned to the control group (GCS scores between 4 and 6). At least four regions of interest (ROIs) from different anatomical regions were measured, the mean transit time (MTT), cerebral blood flow (CBF), and signal intensity time-to-course graphic were calculated. A second neurological examination (including an apnea test) was accepted as the gold standard method for the diagnosis of brain death.

**Results:**

DSC-MR perfusion imaging diagnosed brain death with a specificity of 100% (61/61) and a sensitivity of 86.8% (53/61). A cut-off value of maximum 3.5% decrease in the signal intensity time-to-course graphic was calculated by the Youden’s index and established for the to differentiate brain death from other conditions.

**Conclusion:**

DSC-MR perfusion imaging is a promising tool that may be used as a reliable add-on confirmatory diagnostic test for the brain death.

## Introduction

The irreversible loss of brain and brain stem functions is defined as brain death. Diagnosis of brain death is based on neurological assessments and confirmatory testing. However, there is a high variability between clinical examinations and confirmatory testing [1,2]. When there is uncertainty about the reliability of the neurological examination or the apnea test could not be performed, add-on or complementary tests should be performed to verify brain death. The aim of these complementary tests is to shorten the observation period [3]. The main purpose behind the newly developed diagnostics is measurement of brain perfusion. Tools assessing cerebral perfusion, as reflected by the presence of brain blood flow, are the mainstay of the neurological determination of brain death [4,5,6]. Magnetic resonance (MR) perfusion imaging has become increasingly used in the evaluation of a variety of cerebral pathologies, including tumors, infections, and congenital anomalies. Among the various techniques developed for the evaluation of cerebral perfusion, the dynamic susceptibility contrast (DSC) method has been the most widely used [7,8,9]. However, the clinical role of DSC-MR perfusion imaging in brain death diagnosis has not yet been fully established.

In the present study, we report our experience with DSC-MR perfusion imaging in assessing clinically diagnosed cases of brain death. To the best of our knowledge, we report the first prospective study using DSC-MR imaging as a diagnostic test to confirm brain death in a large cohort.

## Materials and Methods

This study was approved by the local ethics committee. All subjects (first-degree relatives) provided informed consent before the imaging acquisition procedure. The study design was based on the principles of CONSORT and STARD.

### Patient Selection

All subjects were selected from the intensive care unit at a single center to ensure maximum homogeneity among the study and control groups.

A total of 91 subjects underwent DSC-MR perfusion imaging.

All subjects in study and control groups were intubated.

**Figure 1:**
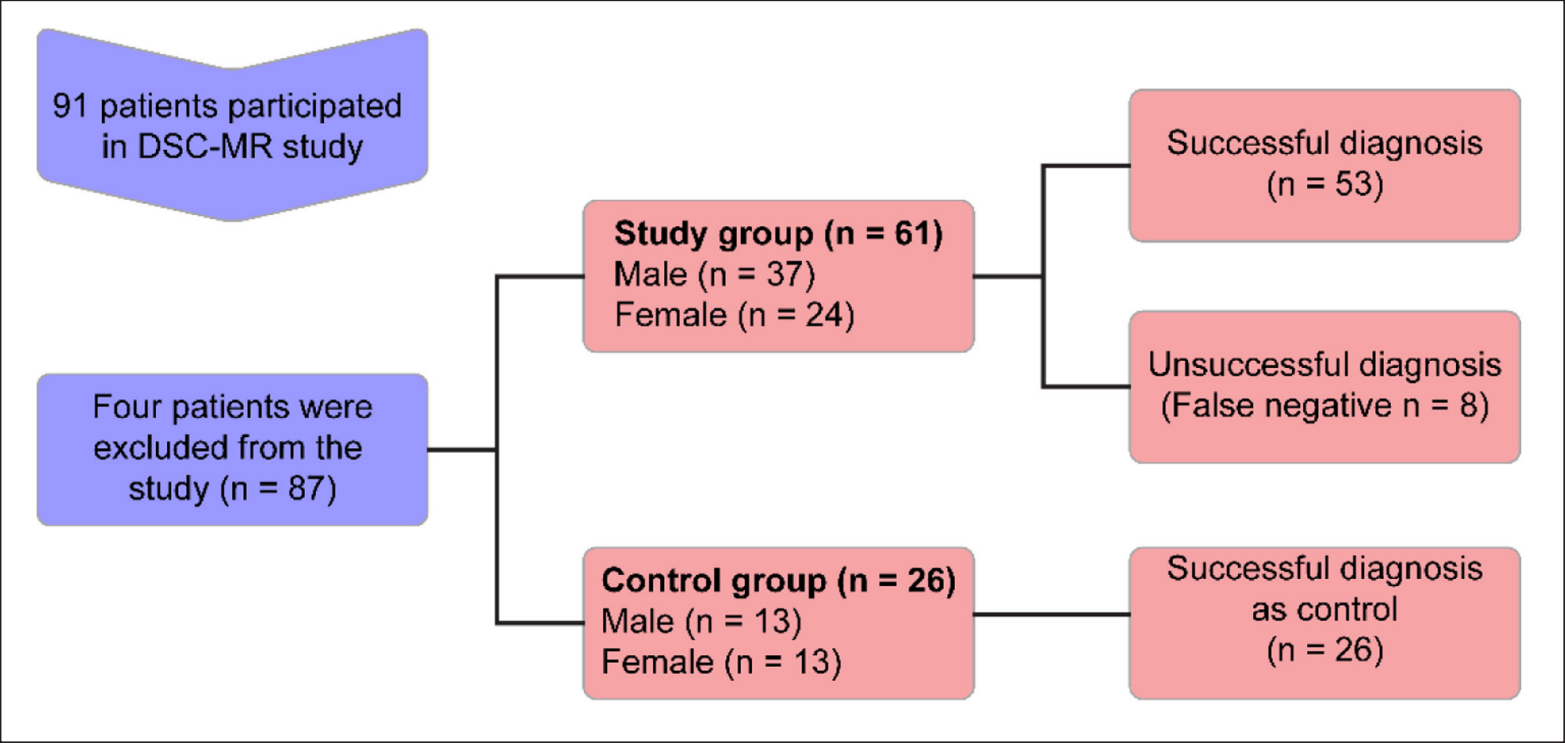
Flow chart of the study.

The subjects were allocated to the groups before the MR examination (Figure 1).

The study group was selected based on positive initial clinical tests for brain death and a Glasgow Coma Scale (GCS) score of 3. The initial clinical tests included:

1. Evaluation of coma status: Subjects were required to have a lack of all evidence of responsiveness.
2. The absence of brain stem reflexes, including oculocephalic and oculovestibular reflexes and pharyngeal or tracheal reflexes, as well as pupils that were fixed midsize or were dilated.
3. The first clinical tests were performed by an 16-year experienced anaesthesiologist. If the first clinical tests suggested a high possibility of brain death, MR perfusion imaging was performed. After MR perfusion imaging (if it revealed brain death), the apnea test, oculovestibular test, and other second clinical tests were performed by an experienced neurologist. Clinical examinations were repeated 12 hours later, and definitive brain death was diagnosed in consensus by the neurologist and anaesthesiologist.

Sixty-one subjects who fulfilled the above criteria were selected as the study group.

### Control Group

The criteria for inclusion in the control group were negative clinical tests for brain death and a GCS score of 3–6 for all patients. Clinical follow-up did not reveal brain death within a period of seven days in 26 patients.

## MR Image Acquisition

All subjects underwent an MR examination 2 to 12 hours after the first clinical indication of brain death (patient group) or 6 to 24 hours after admission (control group).

MR examinations were performed on a 1.5-T scanner (Siemens Magnetom Avanto, Erlangen, Germany) using a standard quadrature birdcage head coil. The DSC-MR perfusion study was performed with echo-echo planar 2D multi-slice imaging using the following parameters: repetition time (TR)/echo time (TE) = 1590/32 ms, slice thickness = 5 mm, slice interval = 0 mm, field-of-view (FOV) = 230 × 230 mm, matrix = 128 × 128. The time interval of the perfusion imaging was 96 seconds (20 slices, 50 images/slice).

A paramagnetic contrast agent (Gadobutrol, Gadovist, Bayer HealthCare, Leverkusen, Germany) was intravenously administered through the antecubital vein with an automatic injector (Mallinckrodt, OptistarElite, St. Louis, USA) at a flow rate of 5 ml/s through an 18- to 20-gauge catheter. A standard dose (0.2 mmol/kg) was administered 5 seconds before the start of image acquisitions, followed by a bolus injection of 20 ml of saline flush.

Routine tests for brain death were part of the imaging protocol at our institute, including namely axial post-contrast (post perfusion weighted imaging) T1-W images (TR: 500 ms, TE: 9 ms) to confirm external carotid artery patency and the time of flight (TOF) MR angiography technique to assess the patency of the external carotid artery and internal carotid artery.

### Image Processing

All perfusion data were transferred to an offline workstation (Leonardo, Siemens, Erlangen, Germany) and processed with a commercially available, dedicated software package (Syngo Via Neuro Perfusion Workflow, Siemens, Erlangen, Germany).

MR perfusion examinations were analyzed by two experienced radiologists (20 years and 18 years of experience in advanced neuroimaging respectively) who were blinded to the clinical assessment and other routine conventional or angiographic MR images. The signal intensity-time curve was obtained from each pixel in the echo planar images. The susceptibility effect of the paramagnetic contrast agent leads to a loss of signal in the signal intensity-time curve. In brain death, the paramagnetic contrast agent cannot reach the intracerebral arteries.

**Figure 2:**
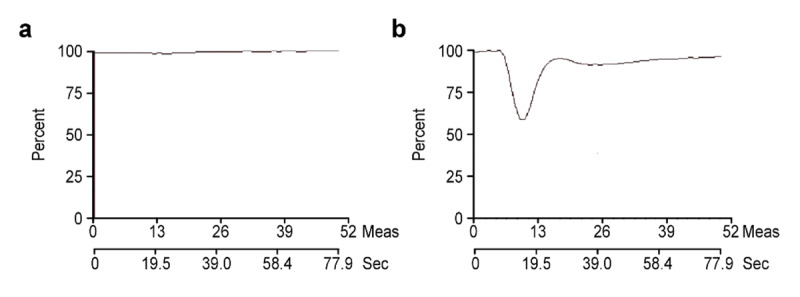
**a.** The graphic of signal intensity-time course absence of signal loss revealed a linear line and accepted as representing brain death. **b.** The graphic of signal intensity-time course, 25–30% depression on the signal intensity-time curve reveals normal cerebral perfusion.

The absence of signal loss revealed by a linear signal intensity-time is generally accepted as a sign of brain death (Figure 2a), whereas a 25–30% depression of the curve stands for normal cerebral perfusion (Figure 2b). We evaluated time-signal intensity curves in four or more regions using circular regions of interest, to detect the abnormalities throughout the brain. These regions included the cerebellum, supratentorial regions such as the frontal and parietal lobes, and the midbrain.

### Statistical Analysis

Kolmogorov-Smirnov test was used to evaluate the normality of the data distribution. Chi-square test was used to determine a relationship between brain death and MR perfusion. We used Youden’s index and the receiver operating characteristic (ROC) curve to estimate the best cut-off values and categorize the time-signal intensity curves. A 3.5% decrease of the time-signal intensity curve was found to be the cut-off value to differentiate brain death from other conditions. The sensitivity, specificity, and positive and negative predictive values were subsequently calculated.

The degree of inter-observer agreement was determined for each imaging test, including perfusion, using weighted e-kappa statistics. Kappa values normally lie between 0 and 1, with 0 indicating agreement purely by chance and 1 indicating perfect agreement. The calculated kappa values ranged from 0.81 to 1.00, indicating very good agreement. Statistical analyses were performed using SPSS software version 21.00 (IBM Corp.). A p-value ≤0.05 was accepted as statistically significant.

## Results

Eighty-seven patients were included in the cohort group; 50 males and 37 females. Sixty-one subjects constituted the study group, and 26 subjects, the control group. The mean age in the brain death group was 55.10 ± 2.197 years, whereas the control group had a mean age of 36.85 ± 2.953 years. The male/female ratio was 37/24 in the study group and 13/13 in the control group. CT and MR imaging findings are summarized in Table 1. Eleven of the brain death patients became donors for both hepatic and renal transplantation.

**Table 1:** Brain injury characteristics in the study population on computed tomography (CT) or magnetic resonance (MR) imaging.

CT or MR Findings	STUDY GROUP	CONTROL GROUP

Subarachnoid haemorrhage (SAH)	10	6
Parenchymal haemorrhage (PH) +SAH	14	3
Intraventricular haemorrhage (IVH)+PH	12	10
Brain tumor	1	1
Ischemic infarct	8	6
Ischmic haemorrhage	7	-
PH + Epidural haemorrhage + Cerebral contusion	4	–
Diffuse Axonal Injury + PH	5	–
**Total**	**61**	**26**

**Figure 3:**
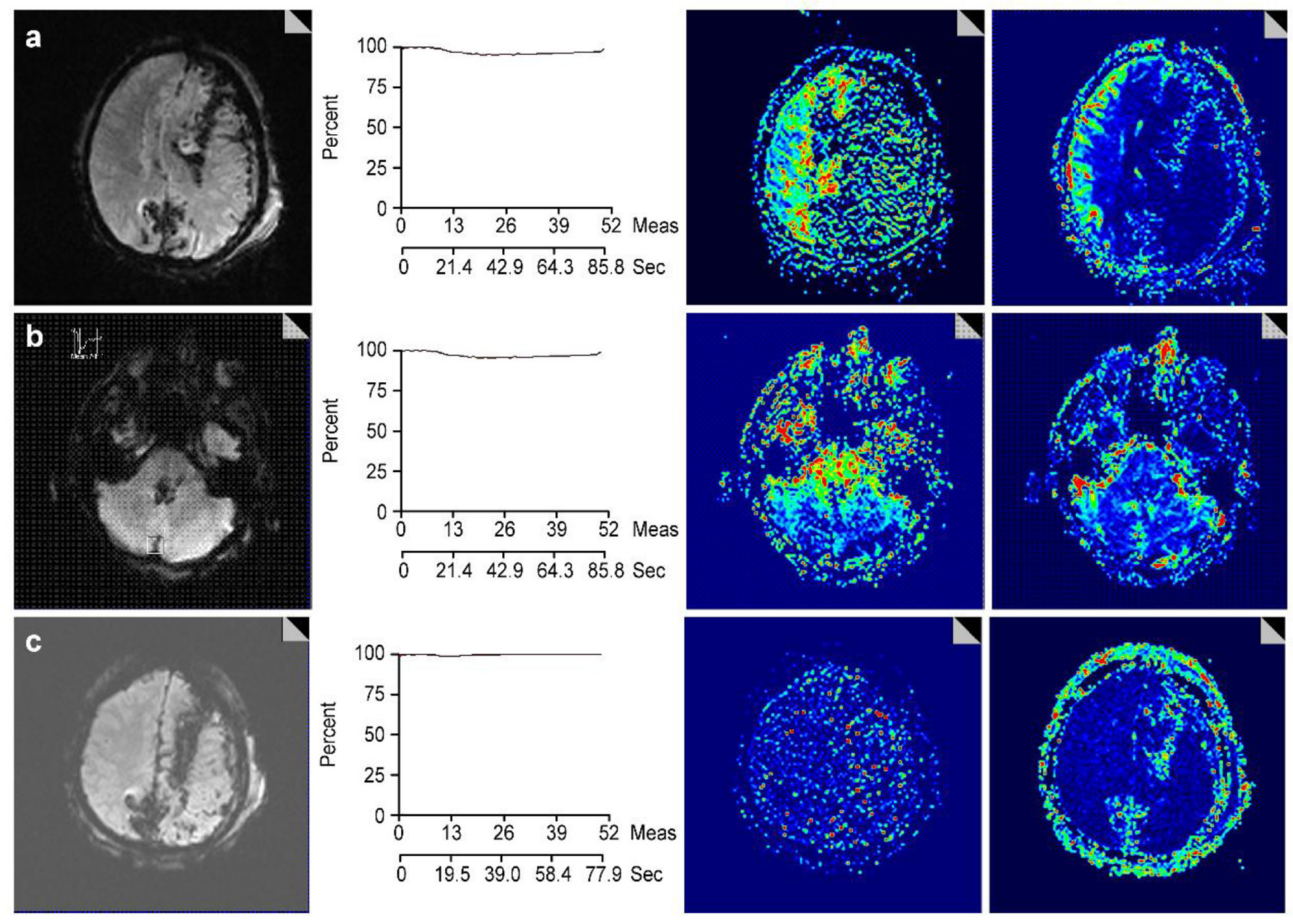
A 28-year-old man was admitted to the intensive care unit after a firearm wound to his left cranium. His Glasgow coma score was 3. **a)** Axial images magnetic resonance images at the level of the centrum semiovale. From left to right panels: The gradient echo MR image shows the hypointense tractus of the bullet in the left cerebral parenchyma and a midline shift to the right. The perfusion signal-time course shows the presence of diminished perfusion. The mean transit time (MTT) and cerebral blood flow (CBF) maps show perfusion in the right cerebral hemisphere and absence of perfusion in the left hemisphere. **b)** Axial images were acquired at the level of the pons in the same order as in a): Both the MTT and CBF maps show the presence of perfusion in the cerebellum and the pons. **c)** A second cerebral MR perfusion examination was performed one day later. Axial images at the level of the centrum semiovale in the same order as in a): The tractus of the bullet in the left cerebral parenchyma remain visible, but the midline shift is less pronounced. The perfusion signal-time course graphic has flattened and both MTT and CBF maps show absence of perfusion in both cerebral hemispheres.

DSC-MR perfusion findings did not correlate with the first neurological tests in eight subjects from the study group as there was normal cerebral perfusion, although the neurological assessment indicated brain death. The reasons for brain death were cerebral parenchymal hemorrhage in two subjects, intraventricular hemorrhage with a large cerebellar parenchymal hemorrhage in four subjects, subarachnoid hemorrhage with intraventricular hemorrhage in one subject, and cerebellar and brain stem infarction due to basilar artery occlusion in one subject. Six of these subjects had a history of intracranial surgery within the preceding 24–73 hours. Five had a ventricular drainage catheter placed to decrease intracranial pressure. These eight subjects underwent repeated MR perfusion imaging 22–34 hours after the first neurological examination. All second MR examinations showed an absence of cerebral perfusion (Figure 3).

DSC-MR perfusion was significantly worse in the brain death group than in the control group (p = 0.008).

**Figure 4:**
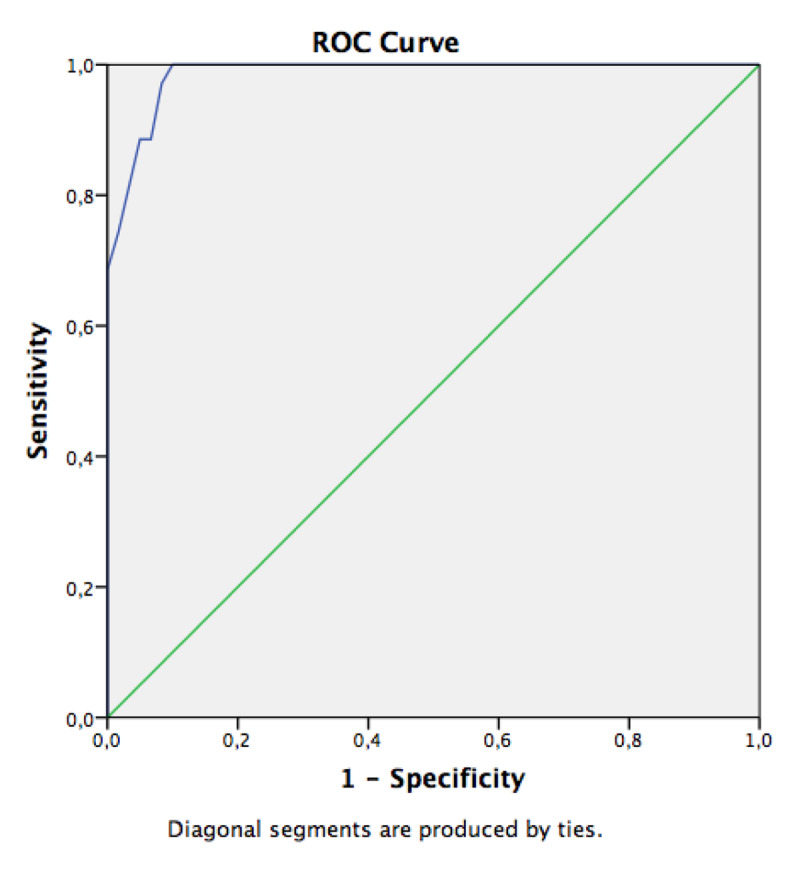
Receiver Operator Characteristics (ROC) of MR perfusion imaging for the diagnosis of brain death. The area under the ROC curve Az is 0.934.

The ROC curve analysis showed that the use of DSC-MR perfusion for the diagnosis of brain death has an area under the curve (Az) of 0.934 (Figure 4).

The sensitivity of the DSC-MR perfusion imaging technique for brain dead was (53/61) 86.8%, and the specificity was (61/61) 100%.

Inter-observer agreement was 0.87 for all observations in the assessment of DSC-MR perfusion in both groups.

## Discussion

We report the first prospective study using DSC-MR imaging as a diagnostic test to confirm brain death in a large cohort. Only one study has been published in the literature in which arterial spin labelling (ASL) was used for MR perfusion in five patients with brain death. The patient selection inherent to this case series did not allow the statistical analysis to investigate the accuracy of ASL in determining brain death [10].

CT perfusion has also been used as a diagnostic tool for assessing brain death. In the English literature, we found two retrospective studies concerning the use of CT perfusion for diagnosis of brain death. Shankar et al. studied CT angiography (CTA) obtained from the CT perfusion for the diagnosis of brain death [11]. This retrospective study, including 11 patients used CTA together with CT perfusion versus the sole use of CTA, revealed that CT perfusion increased the sensitivity. MacDonald et al. used multiple ancillary tests (CT Perfusion, CTA, radionuclide scan) together in diagnosis of brain death [12]. Their results showed that CT perfusion had the highest sensitivity and negative predictive values. We thus believe that using one of the perfusion techniques, either CT or MR, increases the sensitivity for brain death diagnosis as compared to cross-sectional angiographies. However, more prospective randomized control studies are necessary to support this.

We used the signal intensity-time course graphic to assess cerebral perfusion. The interpretation of a semi-quantitative analysis is simple and did not generate inter-observer variability in our study. According to our findings, DSC-MR perfusion imaging successfully revealed the lack of cerebral blood flow in the brain death cohort population using both visual and semi-quantitative analyses. Because the lack of cerebral blood flow is the main ‘gold standard’ consideration for the diagnosis of brain death, DSC-MRI perfusion imaging has potential as a reliable confirmatory test for diagnosing brain death. Among the many factors that make the use of DSC for diagnosing brain death a challenge, the most important factor is that the perfusion parameters based on the DSC reflect only relative values. We analysed the signal intensity-time curve to overcome this drawback and elucidate the clinical role of DSC-MR perfusion imaging.

The optimal diagnostic cut-off value for the decrease in the signal-intensity time-curve was 3.5% and a change between 3.5–25% is suspicious of brain death and requires further evaluation. However further prospective studies are necessary to collect more parameters and confirm this threshold. Eight patients had false negative results. A retrospective analysis revealed that six of these patients had undergone craniotomy and debulking surgery with the placement of a ventricular drainage catheter. These surgical procedures that decreased the intracranial pressure could lead to increased residual brain perfusion and may explain the false negative results [13]. In this group, all second MR examinations showed an absence of cerebral perfusion.

The major limitation of this study is the absence of a correlation with conventional angiography, but this type of study would raise some ethical considerations. Additionally, a DSC-MRI perfusion study requires highly critical patients to be transferred into the MR imaging scanner. Our cohort group consisted of only adult patients; therefore, we were not able to comment on the success of the DSC-MR perfusion imaging technique in paediatric patients. This point should be addressed in future studies. Dynamic contrast-enhanced MR imaging has inaccurate estimation of cerebral blood volume in patients in whom the BBB has been severely disrupted or destroyed [14]. In our study, over half of the patients (38/61) had severe PH with or without IVH, a large parenchymal infarction with ischaemic haemorrhage, and posttraumatic cerebral contusion with PH. We hypothesize that the disruption of the BBB did not have a strong effect on the ability of MR perfusion imaging to assess brain death.

## Conclusions

DSC-MR imaging is a promising tool the diagnosis of brain death. A less than 3.5% drop in the signal-to-time curve after contrast bolus may be used as a discriminative threshold between living and brain dead patients.
